# Solid-State Method Synthesis of SnO_2_-Decorated g-C_3_N_4_ Nanocomposites with Enhanced Gas-Sensing Property to Ethanol

**DOI:** 10.3390/ma10060604

**Published:** 2017-05-31

**Authors:** Jianliang Cao, Cong Qin, Yan Wang, Huoli Zhang, Guang Sun, Zhanying Zhang

**Affiliations:** 1 Henan Key Laboratory of Coal Green Conversion, School of Chemistry and Chemical Engineering, Henan Polytechnic University, Jiaozuo 454000, China; caojianliang@hpu.edu.cn (J.C.); qincongxy@163.com (C.Q.); zhanghuoli@hpu.edu.cn (H.Z.); zhangzy@hpu.edu.cn (Z.Z.); 2 State Key Laboratory Cultivation Base for Gas Geology and Gas Control (Henan Polytechnic University), Jiaozuo 454000, China

**Keywords:** 2-D graphitic carbon nitride, SnO_2_, nanocomposite, ethanol, gas-sensing performance

## Abstract

SnO_2_/graphitic carbon nitride (g-C_3_N_4_) composites were synthesized via a facile solid-state method by using SnCl_4_·5H_2_O and urea as the precursor. The structure and morphology of the as-synthesized composites were characterized by the techniques of X-ray diffraction (XRD), field-emission scanning electron microscopy (FESEM), transmission electron microscopy (TEM), energy dispersive spectrometer (EDS), thermogravimetry-differential thermal analysis (TG-DTA), X-ray photoelectron spectroscopy (XPS), and N_2_ sorption. The results indicated that the composites possessed a two-dimensional (2-D) structure, and the SnO_2_ nanoparticles were highly dispersed on the surface of the g-C_3_N_4_ nanosheets. The gas-sensing performance of the samples to ethanol was tested, and the SnO_2_/g-C_3_N_4_ nanocomposite-based sensor exhibited admirable properties. The response value (Ra/Rg) of the SnO_2_/g-C_3_N_4_ nanocomposite with 10 wt % 2-D g-C_3_N_4_ content-based sensor to 500 ppm of ethanol was 550 at 300 °C. However, the response value of pure SnO_2_ was only 320. The high surface area of SnO_2_/g-C_3_N_4_-10 (140 m^2^·g^−1^) and the interaction between 2-D g-C_3_N_4_ and SnO_2_ could strongly affect the gas-sensing property.

## 1. Introduction

With the development of social industrialization, the leakage and pollution of poisonous gas occur frequently in people’s daily life. It brings a serious threat to human health [1,2,3,4,5]. Hence, the development and research of gas sensors have become urgent work [6]. In the past several years, various metal oxide semiconductors (MOS) materials, such as SnO_2_ [7], ZnO [8], CuO [9], α-Fe_2_O_3_ [10], Co_3_O_4_ [11], MnO_2_ [12], WO_3_ [13], In_2_O_3_ [14], and NiO [15], were used to prepare gas sensors, which possess the outstanding advantages of low cost, controllable size, high-response value, and fast response and recovery time. For example, Yogendra Kumar Mishra et al. successfully prepared a novel ZnO tetrapod network structure, and the fabricated device structures exhibited excellent sensing behaviors toward H_2_ at 400 °C [16]. Hybrid 3-D networks of ZnO-T with Zn_2_SnO_4_ were synthesized using the FTS approach, and the ZnO-T with Zn_2_SnO_4_-based sensor showed the highest response value (S = 29.3) toward CO gas at 275 °C [17]. Aerographite/nanocrystalline ZnO hybrid network materials were prepared and exhibited strong visible light scattering behavior and broadband photo absorption [18]. As is typical of n-type metal oxide semiconductors, SnO_2_ is widely used as a candidate in the gas-sensing field for its wide band gap of 3.6 eV, good chemical stability, and physical properties. However, when taking into consideration their practical application in gas sensing, there are many defects exposed to us. For example, high working temperature, long response and recovery time, and poor stability and aggregation restrict their gas-sensing development. Therefore, many attempts have been made to improve their gas-sensing properties, such as enhancing the specific surface area and the electrical properties by using two-dimensional (2-D) materials [19,20,21,22,23].

Graphene, a representative of 2-D material, has been a focus of scientific research because of its unique property and structure with a unilaminar sp^2^-hybridized carbon atom configuration. In recent years, graphene and reduced graphene oxide (r-GO) have been widely used for gas-sensing investigation due to their large specific surface area and excellent conductivity [24,25,26,27,28,29,30]. Many researchers reported that metal oxide-decorated graphene nanocomposite-based sensors exhibited superior sensibility to different gases [31,32,33,34,35,36,37]. However, as we know, the preparation process of GO and r-GO is complicated and consumptive. Hence, it is necessary to explore a similar novel structure material with graphene.

Recently, graphitic carbon nitride (g-C_3_N_4_) with its graphite-layered structure, which is similar to graphene, has been studied for various applications, including photo degradation and photocatalysis, due to its large specific surface area and high chemical stability [38,39,40,41]. Until now, there are few reports about the application of gas sensors in the presence of g-C_3_N_4_. Zeng et al. successfully prepared a α-Fe_2_O_3_/g-C_3_N_4_ nanocomposite using a facile refluxing method for the cataluminescence sensing of H_2_S [42]. In our previous work, cocoon-like ZnO-decorated graphitic carbon nitride nanocomposites were synthesized, which showed an impressive response toward ethanol [43]. As far as we know, there is no related report about the application of SnO_2_/g-C_3_N_4_-based sensors in the gas-sensing field.

In our study, we synthesized SnO_2_/g-C_3_N_4_-nanocomposites with different mass ratios of SnO_2_ and g-C_3_N_4_ using a facile solid-state method. The gas-sensing properties, including selectivity, stability, and sensitivity of SnO_2_/g-C_3_N_4_ to ethanol, were investigated. As a result, the SnO_2_/g-C_3_N_4_ nanocomposite-based sensor exhibited a higher response value and better selectivity to ethanol than pure SnO_2_ nanoparticles.

## 2. Materials and Methods

### 2.1. Materials

Urea, Tin (IV) chloride pentahydrate (SnCl_4_·5H_2_O, 99.0%), sodium hydroxide (NaOH), and polyethylene glycol 400 (PEG-400) were purchased from Sinopharm Chemical Reagent Co., Ltd. (Beijing, China). All chemicals were used as received without further purification.

### 2.2. Preparation of g-C_3_N_4_

Graphitic carbon nitride (g-C_3_N_4_) was synthesized by pyrolysis of urea in a muffle furnace; 20 g urea was put into an alumina crucible with a cover, then heated to 250 °C within 110 min and kept at 250 °C for 1 h. The further treatment was performed at 350 and 550 °C for 2 h, respectively. The heating rate of the whole reaction was 2 °C·min^−1^. The yellow power (g-C_3_N_4_) was collected. The collected amount of the g-C_3_N_4_ was about 1 g.

### 2.3. Synthesis of the SnO_2_/g-C_3_N_4_ Nanocomposites

SnO_2_/g-C_3_N_4_ nanocomposites were synthesized using a facile solid-state reaction method. In a typical synthesis procedure, 10 wt % 2-D g-C_3_N_4_ in the composites (SnO_2_/g-C_3_N_4_-10) were prepared using the following method. 3.5 g of SnCl_4_·5H_2_O, 0.167 g of g-C_3_N_4_ and 3 mL of PEG-400 were mixed by grinding in an agate mortar. Then, 1.6 g NaOH was slowly added to the mixture, which was ground for another 30 min. An emission of water vapor and heat during the addition of NaOH was observed. The resulting product was separated by centrifuging and washed several times with distilled water and absolute ethanol. Then, the obtained product was dried at 60 °C for 12 h. Finally, the product was ground to powder. SnO_2_/g-C_3_N_4_ nanocomposites with 7 wt % and 13 wt % g-C_3_N_4_-decorated SnO_2_ were also prepared in accordance with this method and marked as SnO_2_/g-C_3_N_4_-7 and SnO_2_/g-C_3_N_4_-13, respectively. For comparison, the same method was used to synthesize pure SnO_2_ nanoparticles in the absence of g-C_3_N_4_.

### 2.4. Characterization

The crystal microstructure of the sample was identified by X-ray diffraction (XRD, Bruker-AXS D8, Bruker, Madison, WI, USA) using Cu Kα radiation with a wavelength of 0.154 nm. X-ray photoelectron spectroscopy (XPS) measurements were taken on a Perkin-Elmer PHI 5600 spectrophotometer (Perkin Elmer Limited, Waltham Mass, Waltham, MA, USA) with Mg Kα (1253.6 eV) radiation. Scanning electron microscope (SEM) images were observed by field-emission scanning electron microscopy (FESEM, Quanta™250 FEG) (FEI, Eindhoven, The Netherlands). Transmission electron microscopy (TEM) analysis was performed on a JEOL JEM-2100 microscope (JEOL, Tokyo, Japan) operating at 200 kV. Thermal gravity and differential thermal analysis (TG–DTA) was carried out on a TA-SDT Q600 (TA Instruments, New Castle, DE, USA) at a heating rate of 10 °C·min^−1^ under an air atmosphere. Nitrogen adsorption–desorption isotherms were obtained on a Quantachrome Autosorb-iQ sorption analyzer (Quantachrome, Boynton Beach, FL, USA). Before carrying out the measurement, the samples were degassed at 150 °C for more than 6 h. The specific surface areas (S_BET_) of the samples were calculated following the multi-point BET (Brunauer-Emmett-Teller) procedure. The pore size distributions were determined from the adsorption branch of the isotherms using the DFT method.

### 2.5. Sensor Fabrication and Measurements

**Figure 1:**
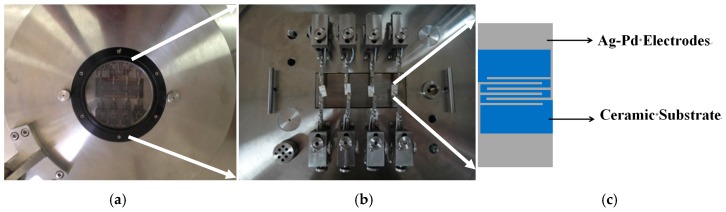
The appearance diagram (**a**) and the internal structure diagram (**b**) of the CGS-4TPS gas-sensing test system, and the structure of the gas sensor substrate (**c**).

The gas-sensing performance of the as-synthesized samples to ethanol was tested using the intelligent gas-sensing analysis system of CGS-4TPS (Beijing Elite Co., Ltd., Beijing, China). Figure 1 shows a brief device schematic diagram. In the process of the gas-sensing test, the relative humidity in the test chamber is 25%. The gas sensors were prepared in a usual way [44]. A small amount of the as-prepared samples were fully ground in an agate mortar with a few drops of ethanol, which served as the agglomerant to form starchiness. Afterwards, the pastes were equably spread on a ceramic substrate (13.4 mm × 7 mm) with interdigitated Ag-Pd electrodes to form the thin film. Before carrying out the test, the substrate was aged at 60 °C for 2 h and at 150 °C for 12 h to improve the stability and repeatability of the gas sensors. The response of the sensors was defined as the ratio of R*_a_*/R*_g_*, where R*_a_* and R*_g_* were the resistances of the sensor measured in air and in test gas, respectively.

## 3. Results and Discussion

### 3.1. Sample Characterization

**Figure 2:**
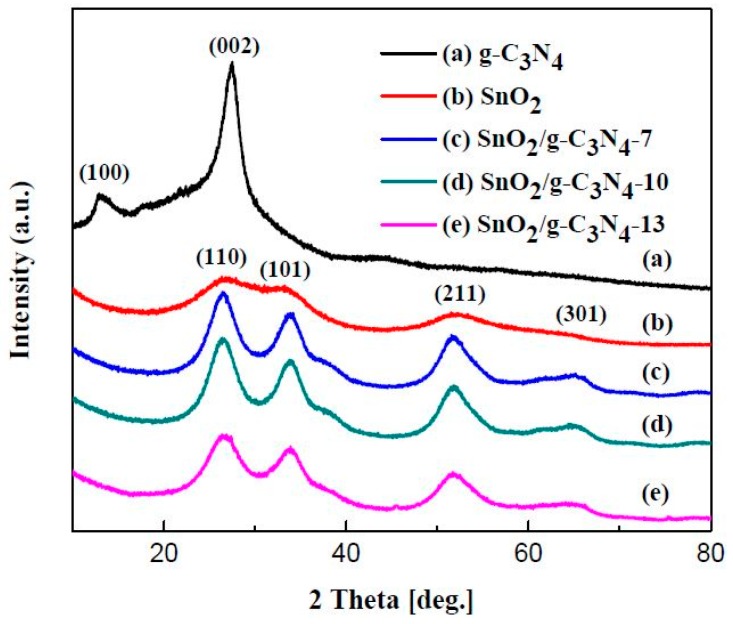
X-ray diffraction (XRD) patterns of the graphitic carbon nitride (g-C_3_N_4_), SnO_2_, and SnO_2_/g-C_3_N_4_ nanocomposites with different g-C_3_N_4_ contents.

Figure 2 shows the XRD patterns of as-prepared g-C_3_N_4_, pure SnO_2_ nanoparticles, and SnO_2_/g-C_3_N_4_ nanocomposites. From Figure 2a, there are two obvious diffraction peaks around 13.1° and 27.5°, which were accorded to the (100) and (002) planes of g-C_3_N_4_. These two peaks are likely to be attributed to the structure of the tri-s-triazine unit with interplanar spacing and the conjugated aromatic system, respectively [39]. It can be concluded that g-C_3_N_4_ was synthesized successfully. As seen from the other curves, there are four distinct diffraction peaks around 26.61°, 33.9°, 51.7°, and 65.9°, which correspond to the (110), (101), (211), and (301) planes of the tetragonal rutile SnO_2_ (JCPDS Card No.41-1445), respectively. However, Figure 2c–e shows that there are no diffraction peaks of g-C_3_N_4_ observed in the curves. This is due to the relatively small content of g-C_3_N_4_ in the nanocomposites or the peak around 27.5° of g-C_3_N_4_ is covered by the peak around 26.61° of SnO_2_.

**Figure 3:**
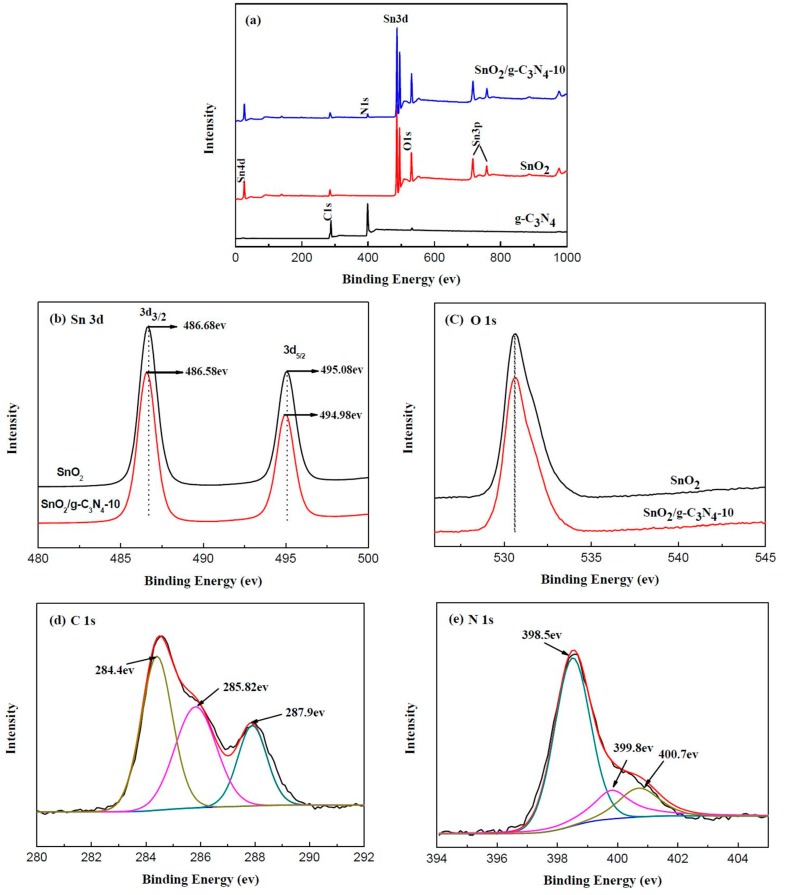
X-ray photoelectron spectroscopy (XPS) survey of g-C_3_N_4_, SnO_2_, and SnO_2_/g-C_3_N_4_-10 samples: (**a**) the general scan spectrum; (**b**) Sn 3d spectrum; (**c**) O 1s spectrum; (**d**) C 1s spectrum; and (**e**) N 1s spectrum.

XPS analysis was carried out to confirm the surface chemical composition and the formation of heterojunction in the SnO_2_/g-C_3_N_4_ sample; the result is shown in Figure 3. Figure 3a displays the survey scan spectra of g-C_3_N_4_, SnO_2_, and SnO_2_/g-C_3_N_4_-10. It is observed that Sn, O, C, and N exist in the SnO_2_/g-C_3_N_4_ composite, and Sn, O, and C exist in SnO_2_. The spectra of g-C_3_N_4_ show only C and N elements. The C 1s peak from SnO_2_ is due to the adventitious carbon. As shown in Figure 3b, two signal peaks of Sn 3d in pure SnO_2_ at binding energies of 486.68 eV and 495.08 eV correspond to Sn 3d_3/2_ and Sn 3d_5/2_, respectively. However, the two signal peaks of Sn 3d in SnO_2_/g-C_3_N_4_-10 had a shade of shift, in which the peak position shifted to 486.58 eV of Sn 3d_3/2_ and 494.98 eV of Sn 3d_5/2_, respectively. This phenomenon can be attributed to the interactions between g-C_3_N_4_ and SnO_2_ and to the heterojunction of the interface region between g-C_3_N_4_ and SnO_2_. For the high-resolution XPS spectra shown in Figure 3c, there are few distinctions of O 1s between SnO_2_ and SnO_2_/g-C_3_N_4_-10. Figure 3d displays the high-resolution XPS spectra of C 1s. The three signal peaks for the C 1s binding energies exist at 284.4, 285.82, and 287.9 eV, respectively. As is well known, the signal at 284.4 eV corresponds to sp^2^ C–C bonds, while the signal at 285.82 eV is identical to the combination of C–N groups. And the signal at 287.9 eV comes from the sp^2^ C atoms from the aromatic rings N–C=N. As is seen in Figure 3e, there are three signals with binding energies at 398.5, 399.8, and 400.7 eV, respectively. The peak at 398.5 eV is ascribed to sp^2^-hybridized aromatic N bonded to C atoms, and the peak at 399.8 eV comes from the tertiary N bonded to C atoms in the form of N–(C)_3_. The peak at 400.7 eV is related to the N–H structure. From the above analysis, the interactions between Sn and g-C_3_N_4_ enhance the electrical conductivity of the nanocomposite, which could be of benefit for the gas-sensing performance.

**Figure 4:**
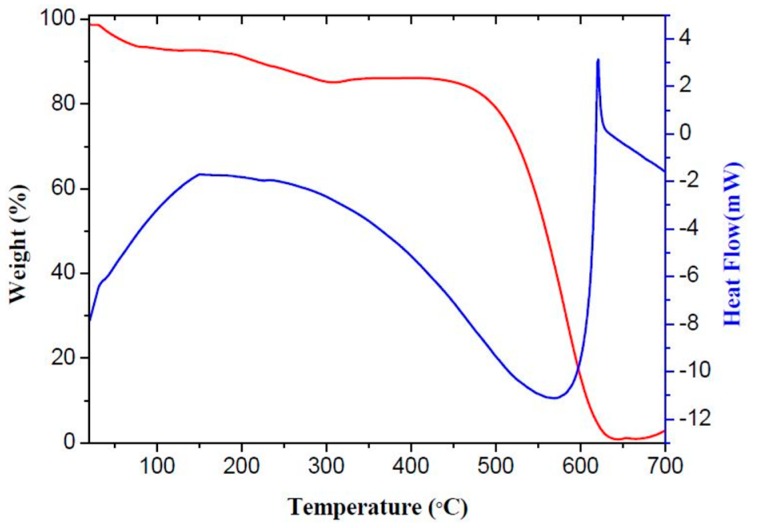
Thermogravimetry–differential thermal analysis (TG–DTA) profiles of g-C_3_N_4_.

TG-DTA analysis was carried out to reveal the weight change situation of g-C_3_N_4_. The temperature range was from room temperature to 700 °C, and the heating rate was 10°/min. As is shown in Figure 4, the first peak was between 100 °C and 300 °C, which is due to the desorption of moisture and solvent. The second peak was between 400 °C and 600 °C, which is due to the combustion of g-C_3_N_4_ in air. This result demonstrates that g-C_3_N_4_ was not decomposed at the optimum temperature of 300 °C in the process of testing for gas-sensing properties.

**Figure 5:**
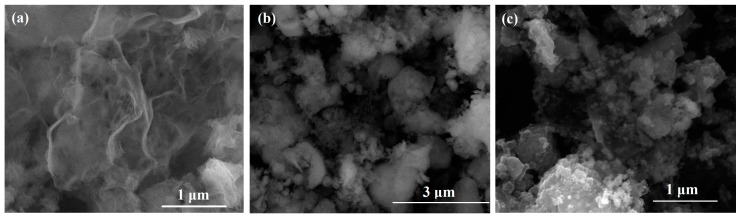
Scanning electron microscope (SEM) images of (**a**) g-C_3_N_4_; (**b**) SnO_2_; and (**c**) SnO_2_/g-C_3_N_4_-10 samples.

The SEM images of g-C_3_N_4_, SnO_2_, and SnO_2_/g-C_3_N_4_ composite are shown in Figure 5. Figure 5a displays the SEM image of g-C_3_N_4_. On the edge of the thin layers, many wrinkles can be clearly seen, which are representative of 2-D materials. Figure 5b shows many SnO_2_ nanoparticles agglomerated together with different size. As shown in Figure 5c, plenty of particles are highly decentralized on the g-C_3_N_4_ sheets. This could be beneficial to improving the gas-sensing properties.

**Figure 6:**
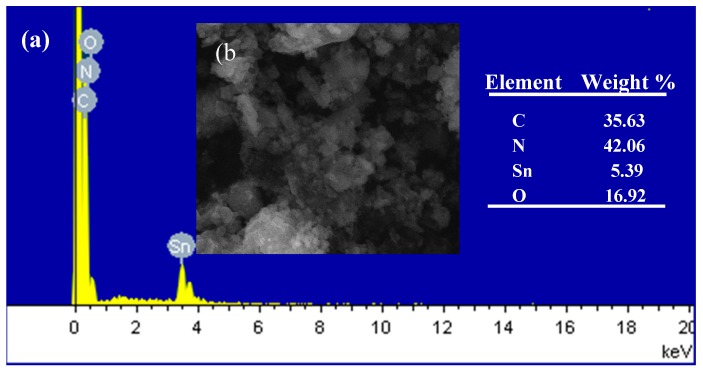

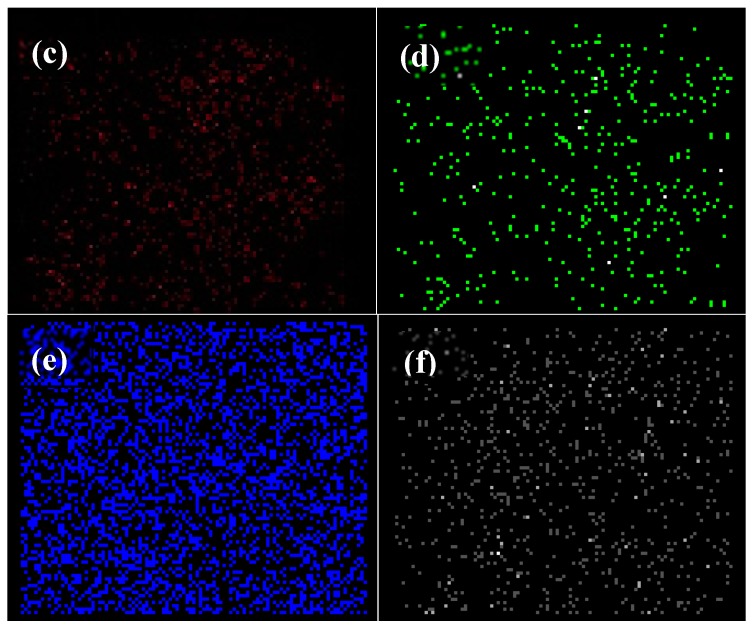
Energy dispersive spectrometer (EDS) spectra (**a**) and SEM image (**b**) of the SnO_2_/g-C_3_N_4_-10 nanocomposite, and EDS mappings of the Sn (**c**), O (**d**), C (**e**), and N (**f**) element related to (**b**).

Figure 6a displays the typical spectra of SnO_2_/g-C_3_N_4_-10 composite recorded from the surface area that was observed in Figure 6b, where the peaks of Sn, O, C, and N are simultaneously existent. The percentage composition of the four elements of C, N, Sn, and O is 35.63 wt %, 42.06 wt %, 5.39 wt %, and 16.92 wt %, respectively. The energy dispersive spectrometer (EDS) mapping of the four elements Sn, O, C, and N are shown in Figure 6c, Figure 6d, Figure 6e, and Figure 6f, respectively. The distributions of these four elements are clearly observed. According to Figure 6, the structural feature of the SnO_2_/g-C_3_N_4_-10 composite is that 2-D g-C_3_N_4_ and SnO_2_ particles are effectively combined. It can be concluded that the SnO_2_/g-C_3_N_4_ composites were synthesized successfully using the solid-state method, which is applicable to large-scale production.

**Figure 7:**
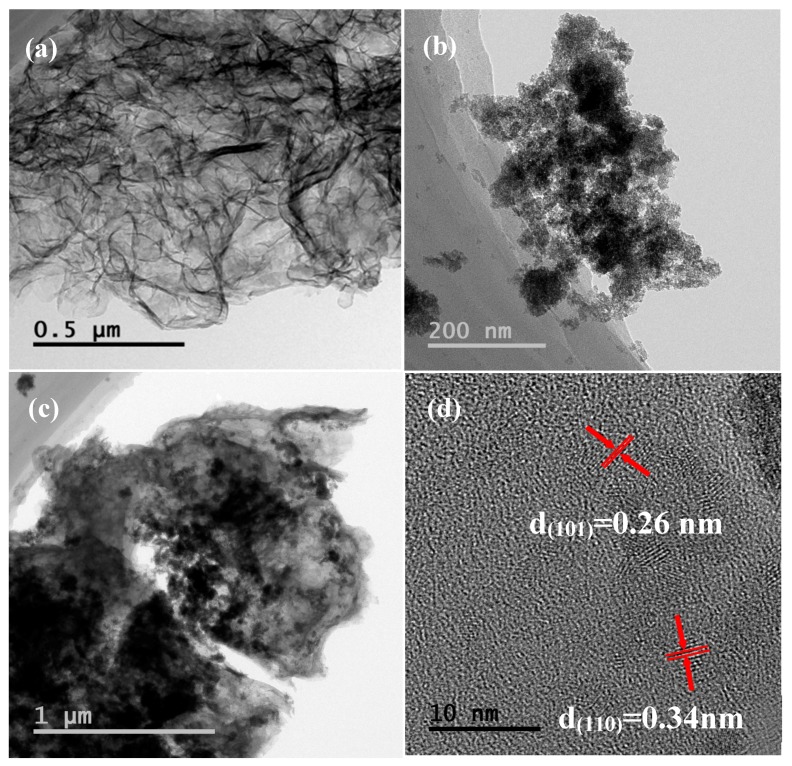
Transmission electron microscopy (TEM) images of (**a**) g-C_3_N_4_; (**b**) SnO_2_; and (**c**) SnO_2_/g-C_3_N_4_-10; and (**d**) HRTEM image of the SnO_2_/g-C_3_N_4_-10 composite.

Figure 7 shows the TEM and HRTEM images of g-C_3_N_4_, SnO_2_ and of the SnO_2_/g-C_3_N_4_ nanocomposite. As shown in Figure 7a, it can be seen that there are plenty of gauffers in the floccules. Figure 7b shows that the pure SnO_2_ samples consist of many nanoparticles. Meanwhile, as can be seen from Figure 7c, the SnO_2_ nanoparticles are highly dispersed on the surface of g-C_3_N_4_. From Figure 7d, the lattice fringes with interplanar spacings of 0.26 and 0.34 nm can be assigned to the (101) and (110) planes of the g-C_3_N_4_-supported SnO_2_ nanoparticles.

**Figure 8:**
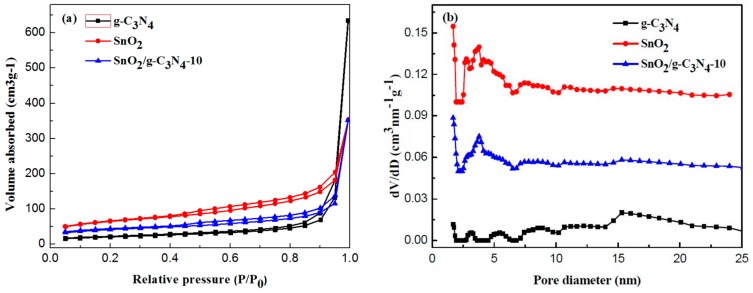
(**a**) N_2_ adsorption–desorption isotherms and (**b**) the corresponding pore size distribution curves of the g-C_3_N_4_, SnO_2_, and SnO_2_/g-C_3_N_4_-10 samples. The dV/dD value was shifted by 0.05 and 0.1 units for the curves of data sets SnO_2_/g-C_3_N_4_-10 and SnO_2_, respectively.

Figure 8 depicts the N_2_ adsorption–desorption isotherms and the corresponding pore size distribution of the as-prepared g-C_3_N_4_, SnO_2_, and SnO_2_/g-C_3_N_4_-10 samples. It can be seen from Figure 8a that the isotherms of the three samples show type IV, which is the typical characteristic of mesoporous material according to the IUPAC. The well-defined hysteresis loop of the SnO_2_/g-C_3_N_4_-10 sample belongs to the H_3_-type, indicating the presence of an aggregation of laminated structure with narrow slits formed by g-C_3_N_4_ and SnO_2_ nanoparticles. The corresponding pore size distributions of these three samples are shown in Figure 8b. It can be clearly seen that the pore diameters of SnO_2_ and SnO_2_/g-C_3_N_4_-10 are relatively small, and the majority concentrate upon about 2 nm according to the DFT method. The BET-calculated results show that the specific surface areas of g-C_3_N_4_, SnO_2_, and SnO_2_/g-C_3_N_4_-10 samples are 60.7 m^2^·g^−1^, 173.2 m^2^·g^−1^, and 140.0 m^2^·g^−1^, respectively. The high specific surface area could be in favor of enhancing gas-sensing properties.

### 3.2. Gas-Sensing Property

**Figure 9:**
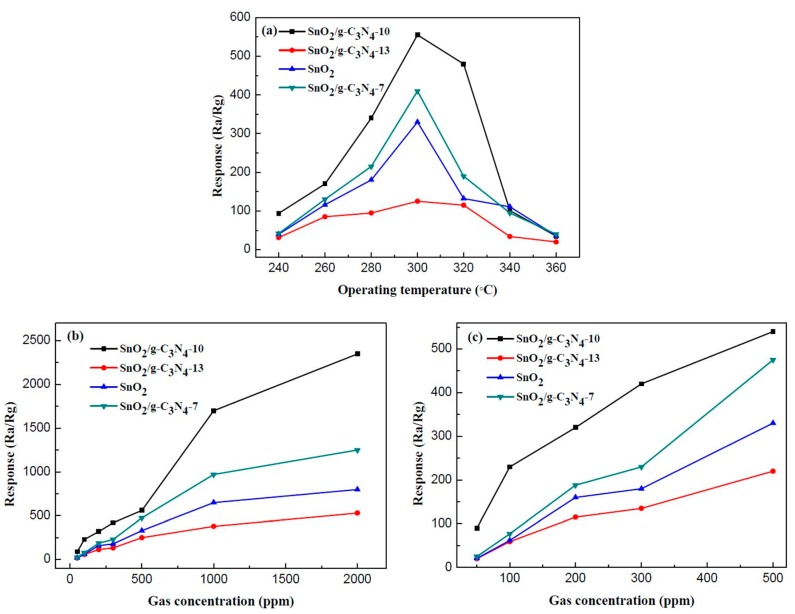
(**a**) Response values of the sensors based on SnO_2_, SnO_2_/g-C_3_N_4_-7, SnO_2_/g-C_3_N_4_-10, and SnO_2_/g-C_3_N_4_-13 to 500 ppm of ethanol as a function of operating temperature; (**b**,**c**) the responses of sensors (SnO_2_, SnO_2_/g-C_3_N_4_-7, SnO_2_/g-C_3_N_4_-10, and SnO_2_/g-C_3_N_4_-13) operated at 300 °C versus different concentrations of ethanol.

The gas-sensing properties of the as-prepared samples to ethanol vapor were investigated, in detail. Figure 9a shows the response values of pure SnO_2_ and SnO_2_/g-C_3_N_4_-based sensors to 500 ppm of ethanol at different operating temperatures. It can be clearly observed that the response values increased with the increase of the operating temperature. However, the response values decrease when the temperature is above 300 °C. The maximum response of SnO_2_/g-C_3_N_4_-10 is R*_a_*/R*_g_* = 555 at 300 °C, which is much higher than that of the pure SnO_2_-based sensor. It reaches the maximum response when the mass percentage of g-C_3_N_4_ in the composites is 10%. From the curves, the response value of SnO_2_/g-C_3_N_4_-13 sample is lower than that of the pure SnO_2_-based sensor. The high content of g-C_3_N_4_ may lead to the connection of the g-C_3_N_4_ nanosheets, which could form the micro-electric bridges on the surface. The micro-electric bridges may result in the semiconductor’s resistance being reduced. Figure 9b,c display the response values of the four samples (SnO_2_, SnO_2_/g-C_3_N_4_-7, SnO_2_/g-C_3_N_4_-10, and SnO_2_/g-C_3_N_4_-13) at 300 °C to different concentrations of ethanol. As shown in the curves, the response values increased with increasing ethanol concentrations. The slope of the curves increased rapidly when the concentration range of ethanol was from 50 ppm to 500 ppm. However, it increased slowly with increasing concentrations in the range of 500–2000 ppm. It can be concluded that the adsorption to ethanol has approached saturation value when the concentration reaches 2000 ppm. To evaluate the gas-sensing performances of SnO_2_/g-C_3_N_4_ composite, the comparison between this work and other literature is summarized in Table 1. As can be observed, the SnO_2_/g-C_3_N_4_ composite exhibits superior performances compared with other SnO_2_-based sensors.

**Figure 10:**
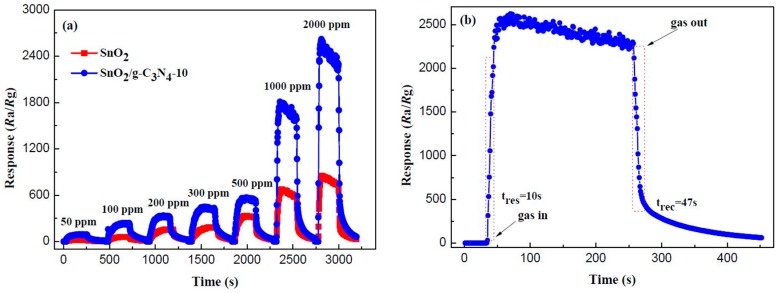
(**a**) Real-time response curves of pure SnO_2_ and SnO_2_/g-C_3_N_4_-10 to ethanol in the range of 500–2000 ppm, and (**b**) response–recovery curve of SnO_2_/g-C_3_N_4_-10 to 2000 ppm of ethanol.

Figure 10a displays the real-time response curves of the pure SnO_2_ and SnO_2_/g-C_3_N_4_-10 to ethanol in the range of 50–2000 ppm at 300 °C. As shown in the curves, the response values of the both sensors increased with the increasing concentration of ethanol in the range of 50–2000 ppm. The response value of the SnO_2_/g-C_3_N_4_-10-based sensor is much higher than that of the pure SnO_2_-based sensor to the same concentration of ethanol. The response values of pure SnO_2_ and SnO_2_/g-C_3_N_4_-10 to 2000 ppm of ethanol are 800 and 2400, respectively. The response–recovery time curve of SnO_2_/g-C_3_N_4_-10 to 2000 ppm of ethanol is shown in Figure 10b. It can be clearly observed that the response increased and decreased promptly when the SnO_2_/g-C_3_N_4_-10-based sensor was exposed to and separated from ethanol, respectively. The response time and the recovery time are 10 s and 47 s, respectively. The relatively rapid response and recovery time could be due to the unique structure of the 2-D g-C_3_N_4_-supported SnO_2_ nanoparticles.

**Figure 11:**
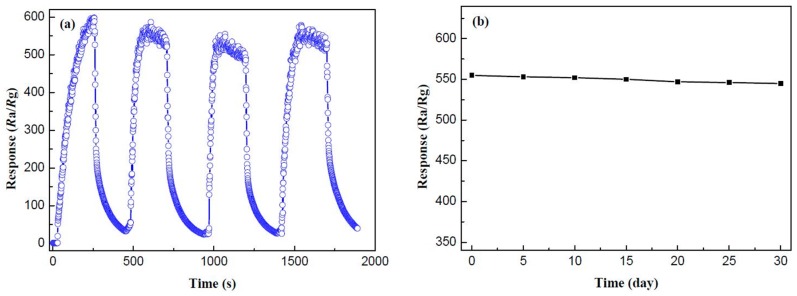
(**a**) Repeatability and (**b**) stability measurements of the SnO_2_/g-C_3_N_4_-10 sensors to 500 ppm of ethanol at 300 °C.

Repeatability and stability are both crucial influence factors of gas-sensing properties. Figure 11a reveals the repeatability of the SnO_2_/g-C_3_N_4_-10 sensor to 500 ppm of ethanol at 300 °C. As shown in the curves, the response values of the four response–recovery cycles are almost the same, namely 570, 565, 554, and 566, respectively. It can be concluded that the as-prepared SnO_2_/g-C_3_N_4_-10 sensor has an admirable repeatability for ethanol gas sensing. A durable response value was measured to explore the stability of the SnO_2_/g-C_3_N_4_-10 sensor. Figure 11b displays the test result for every five days, and the response values to 500 ppm of ethanol at 300 °C are maintained around 550. Hence, the conclusion may be drawn that the SnO_2_/g-C_3_N_4_-10-based sensor has an unexceptionable stability for ethanol gas sensing.

**Figure 12:**
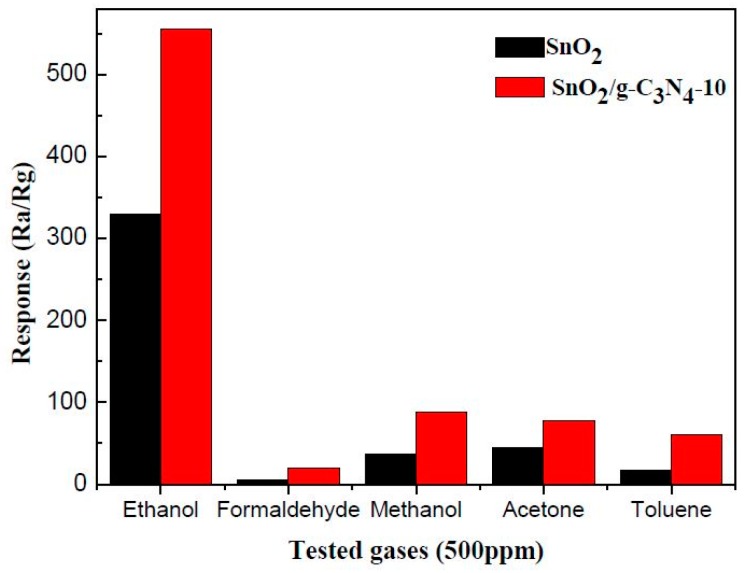
Responses of SnO_2_ and SnO_2_/g-C_3_N_4_-10-based sensors to 500 ppm of different reducing gases at 300 °C.

It is well known that selectivity is another key criteria for measuring the quality of gas sensors. Figure 12 shows the selectivity test results of the pure SnO_2_ and SnO_2_/g-C_3_N_4_-10 sensors to five different gases of 500 ppm, including methanol, ethanol, toluene, formaldehyde, and acetone. It can be seen that the SnO_2_/g-C_3_N_4_-10 sensor has a selectivity to ethanol superior to that of other gases compared to the pure SnO_2_ sensor at 300 °C. The higher response to ethanol may be because ethanol is more likely to lose electrons in the process of a redox reaction with the absorbed oxygen, and the hydroxyl group (–OH) is much easier to oxidize at the optimum operating temperature.

**Figure 13:**
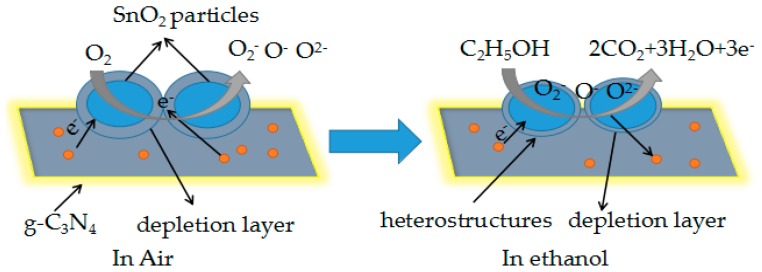
Schematic diagram of test gas reaction with the as-prepared nanocomposite.

As is well known, SnO_2_ is a typical n-type metal oxide semiconductor, and there are several different types of gas-sensing mechanisms. Generally, the surface-controlled type can be used to explain the mechanism of the SnO_2_/g-C_3_N_4_ composite towards ethanol. The resistance changes when the sensor is exposed to different types of gases. When the sensor was exposed in air, oxygen molecules would adsorb on the surface of SnO_2_ and capture electrons from the conduction band of SnO_2_. Then, oxygen molecules were ionized to O^2−^, O^−^, and O_2_^−^, and the formation of depletion layers led to an increase in resistance of the composite sensor. However, when the sensor was exposed to the ethanol gas under high temperature, the ethanol molecules would react with oxygen ions absorbed on the surface of the sensor. As a result, the ethanol molecules were oxidized into acetaldehyde and eventually oxidized into carbon dioxide and water. The trapped electrons were released back to the depletion layer of the sensing film, resulting in a decrease in the resistance of the composite-based sensor, as is shown in Figure 13.

The SnO_2_/g-C_3_N_4_ composites exhibit better gas-sensing properties than pure SnO_2_ nanoparticles. In this nanocomposite, g-C_3_N_4_ served as a support and stuck to SnO_2_ nanoparticles. This support can prevent the aggregation of SnO_2_ nanoparticles. Consequently, this unique structure with large specific surface area is beneficial to the mass of oxygen molecules adsorbed on to the surface of SnO_2_ and to the adsorption and diffusion of ethanol molecules, leading to an enhanced reaction between ethanol gas molecules and oxygen anions. Beyond that, the improved gas-sensing performances may also be attributed to the heterojunction of the interface region between g-C_3_N_4_ and SnO_2_ and to the interactions between Sn and g-C_3_N_4_ verified in the XPS results. The electrical property at the heterojunction changes when ethanol gas molecules pass through the interface region between g-C_3_N_4_ and SnO_2_. Both SnO_2_ and g-C_3_N_4_ are n-type semiconductors. The band gaps are 3.71 eV and 2.7 eV, respectively. The conduction band level of g-C_3_N_4_ is more negative than that of SnO_2_. When SnO_2_ and g-C_3_N_4_ were combined, they formed a heterojunction structure. The electrons will inflow from the conduction band of g-C_3_N_4_ to the conduction band of SnO_2_, leading to a higher potential barrier. As a result, the electrons and holes are separated. Meanwhile, the heterojunction structure may suppress the recombination of the electron–hole pair and urge electrons to quickly transfer from the ethanol vapor to the surface of SnO_2_/g-C_3_N_4_. Therefore, this leads to a higher response because of the increased conductivity of the heterojunction structure [49].

## 4. Conclusions

In summary, we demonstrated an ethanol gas sensor based on a SnO_2_/g-C_3_N_4_ nanocomposite, which was synthesized by a facile solid-state method using a grinding treatment at room temperature. The SnO_2_ nanoparticles were highly distributed on the g-C_3_N_4_ sheets. The gas-sensing properties of the SnO_2_/g-C_3_N_4_ nanocomposite-based sensors exhibited enhanced gas-sensing properties compared to pure SnO_2_, including sensitivity and selectivity. The ameliorative sensitivity may be due to the large specific surface area and the interaction between 2-D g-C_3_N_4_ and SnO_2_ nanoparticles. As a result, the SnO_2_/g-C_3_N_4_ nanocomposite is a promising candidate for high-performance ethanol gas-sensing application.

## Figures and Tables

**Table 1:** Comparison of the performance of various SnO_2_-based gas sensors toward ethanol.

Sensing Materials	Ethanol Concentration (ppm)	Temperature (°C)	Response (Ra/Rg)	Reference
RGO-SnO_2_	100	300	70	[45]
Ni-doped SnO_2_	100	260	30	[46]
Fe_2_O_3_/SnO_2_	100	300	30	[47]
Au/SnO_2_	150	340	30	[48]
SnO_2_/g-C_3_N_4_-10	100	300	230	this work
